# Histomorphological and Histochemical Observations of the Common Myna (*Acridotheres tristis*) Tongue

**DOI:** 10.1155/2013/980465

**Published:** 2013-01-15

**Authors:** Khalid Kamil Kadhim, AL-Timmemi Hameed, Thamir A. Abass

**Affiliations:** ^1^Department of Anatomy and Histology, Faculty of Veterinary Medicine, University of Baghdad, Baghdad, Iraq; ^2^Department of Surgery and Obstetrics, Faculty of Veterinary Medicine, University of Baghdad, Baghdad, Iraq

## Abstract

Common myna tongue was studied histomorphologically and histochemically. Four tongues of adult birds were carried out macroscopically and microscopically. The tongue was triangular; the dorsum of the body had median groove. Two to three backward directed papillae were located on each side of the body-base junction. A single transverse row of pharyngeal papillae was located behind the laryngeal cleft. The parakeratinized mucosa covered the entire surface of the tongue except clearly keratinized band on the ventrolateral surface and the conical papillae. Compared with the lateral group (LG), the secretory cells of the medial group (MG) of the anterior lingual glands (ALG) and the posterior lingual glands (PLG) contained large amount of mucin. It was neutral mucin. However, the LG had weak acid mucin with carboxylated group. Meanwhile, the MG of the ALG and the PLG had strong acid mucin with both carboxylated and sulphated groups. In conclusion, the morphological observation of the common myna tongue showed some variation from the other birds. Histochemical results indicated the differences between the LG and MG of the anterior lingual glands. However, no difference was observed between the latter and the PLG.

## 1. Introduction

Common myna is omnivorous bird native to Asia [1]. The tongue of different species of birds has been studied, on little tern [2], goose [3], eagle [4], and ostrich [5] and in red jungle fowl [6]. The conclusions of these studies proved that the tongue was modified according to the method of food intake, type of food, and habitat. The tongue has provided by conical papillae that are arranged in transverse row in chicken [7], in common kestrel [8], and in red jungle fowl [6]. However, in goose tongue, these conical papillae are located in the midline between the lingual body and radix. The pharyngeal papillae of the red jungle fowl are arranged in one transverse row [6], and double rows in chicken [7]. The root and dorsum tongue are covered by parakeratinized epithelium [4]. The tongue of the herbivorous and granivorous birds is covered with thick keratinize mucosa [2, 5]. However, the keratinization is lesser in the tongue of the water habitat birds [9, 10]. The lingual salivary glands of different types of birds have been described [6, 11, 12]; it has been shown that the lingual salivary glands produce neutral and sulphated mucin. There is a dearth of information regarding this wild bird (common myna) particularly the morphology of the tongue; therefore, this study was conducted to add information regarding the anatomy and classification of the mucin in the tongue of this bird.

## 2. Materials and Methods

Four adult birds were used in this experiment. The birds were captured from the village southern Serdang, Selangor, Malaysia. The birds were dissected after capitis dislocation. The tongue was washed with normal saline solution and then fixed in 10% neutral buffered formalin. The macroscopical examination of the external surface of the tongue was carried out using stereomicroscope image analysis (SMZ 1500 digital camera). For histological and histochemical examinations, paraffin sections (5 *μ*m) were cut from the tongue and stained with routine hematoxylin and eosin stain, PAS stain for vicinal dial group of mucin [13], alcian blue pH 1 and pH 2.5 for strong and weak acid mucin, respectively, alcian blue-PAS stain for acid and neutral mucin, aldehyde fuchsin-alcian blue technique for sulphate and carboxylated acid mucin [14].

## 3. Results

### 3.1. Gross Findings

**Figure 1:**
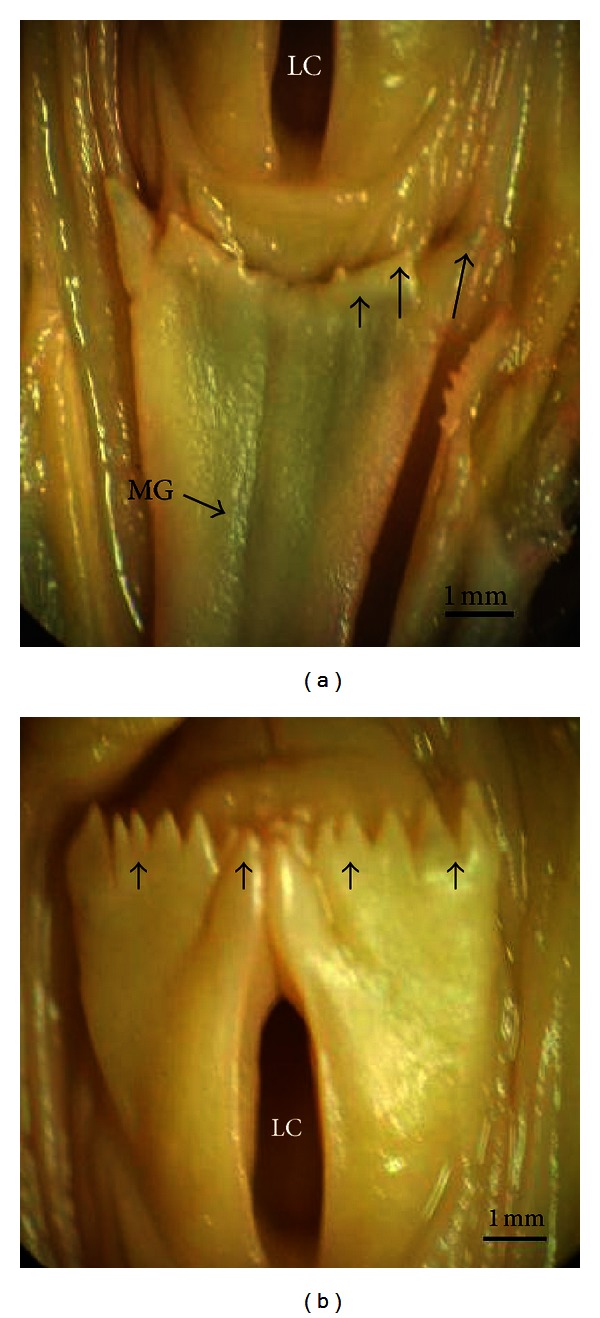
Macrophotographs of the common myna tongue. (a) Dorsum tongue with median groove (MG), lingual papillae (arrows), and laryngeal cleft (LC). (b) Tongue base with a single row of pharyngeal papillae (arrows) behind the laryngeal cleft (LC).

The common myna tongue has triangular shape and occupied the cavity of the lower beak. The dorsum had a median groove extended from the tip to the body-root junction. Whereas, the ventrolateral surfaces of the tongue seemed too hard. At least two to three lingual conical papillae were directed backward and are arranged transversely in each side on the body-base junction (Figure 1(a)).

However, there was a single row of pharyngeal papillae that are arranged transversely behind the laryngeal cleft (Figure 1(b)).

### 3.2. Histological and Histochemical Observations

**Figure 2:**
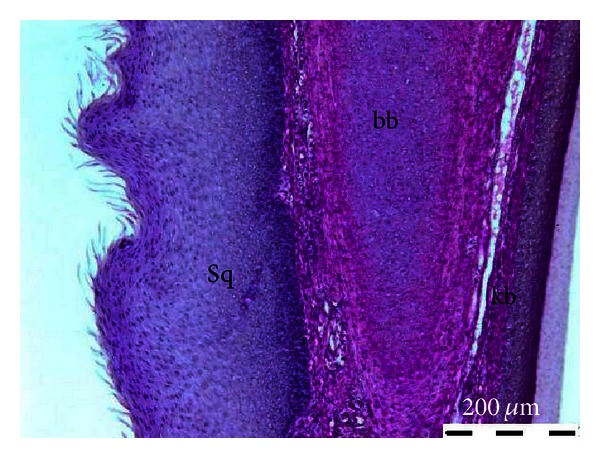
Microphotograph of the common myna tongue (longitudinal section) showing the dorsum with thick stratified squamous epithelium (Sq), base hyoid bone (bb), and the keratinized ventrolateral band (kb). H&E.

The mucous membrane that lines the tongue is composed of stratified squamous epithelium with varying degrees of keratinization; it was thickest on the dorsum of the tongue, but with a thin parakeratinized layer of the stratum corneum. However, this layer showed highly keratinized band in the ventrolateral and pharyngeal conical papillae (Figure 2).

**Figure 3:**
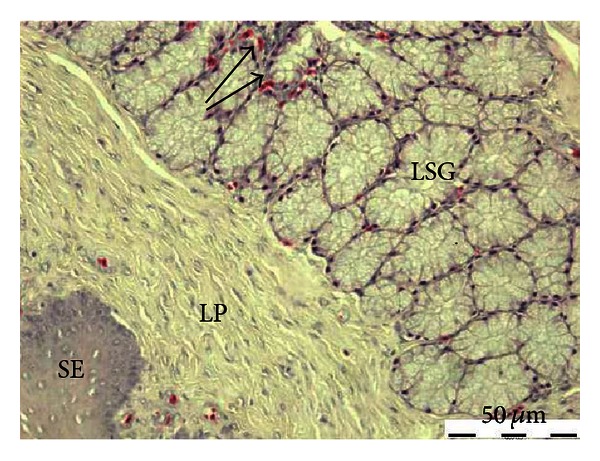
Microphotograph of the common myna tongue showing the stratified squamous epithelium (Sq), lamina propria (LP), and the lingual salivary glands (LSG). The acini are surrounded by blood vessels (arrows). H&E.

The lingual salivary glands located beneath the surfaces of the tongue. The keratinization of the dorsum tongue was restricted on the lingual mucosa, dorsolateral to the basihyal bone at the posterior half of the free part of the tongue (anterior lingual glands), and in the dorsal surface of the tongue base (posterior lingual glands). The acinar secretory units are lined by columnar cells with basal nuclei (Figure 3). The apex of these cells was filled with secretory granules. However, the amount of mucin in the cytoplasm of the glandular cells showed some difference; the medial group of the anterior lingual glands and the posterior lingual glands contained more mucin granules than the lateral group of the anterior lingual glands. The connective tissue that surrounds the glandular acini was rich in blood vessels (Figure 3). The body of the tongue was supported by basihyal bone and the extrinsic muscles. The anterior part of the tongue was free from the glandular tissue.

**Figure 4:**
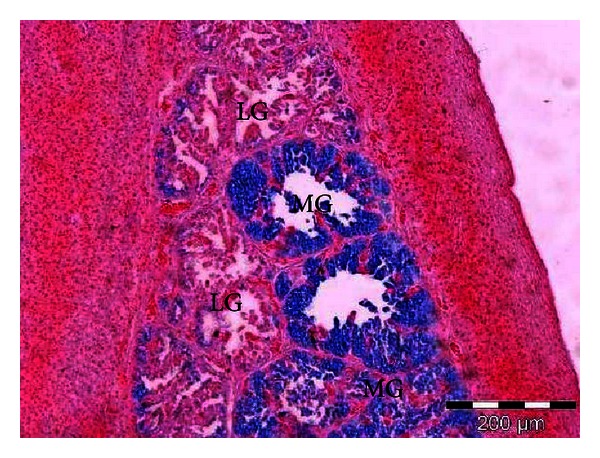
Microphotograph of the interior lingual glands of the common myna tongue, showing weak acid mucin reaction in the lateral group (LG) and moderate acid mucin reaction in the medial group (MG). Alcian blue (pH 1).

**Figure 5:**
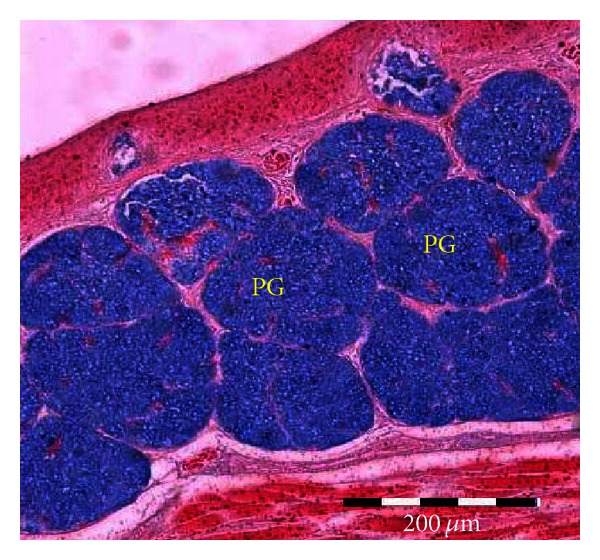
Microphotograph of the posterior lingual glands (PG) of the common myna tongue, showing strong acid mucin reaction. Alcian blue (pH 2.5).

Histochemically, the mucin granules of the secretory cells were PAS positive; however, the MG and the PLG showed more mucin reaction than the LG. Staining with alcian blue (pH 1), the LG stained weakly, while the MG and the PLG stained moderately. When the pH increased to 2.5, the intensity of mucin to the stain increased moderately in the LG. however, it is strongly reacted in the MG and the PLG (Figures 4 and 5).

**Figure 6:**
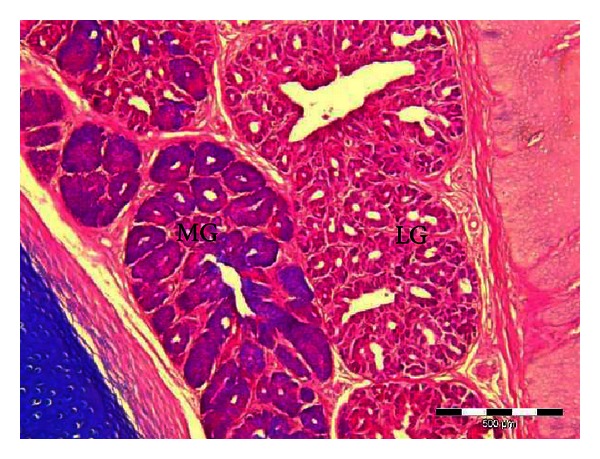
Microphotograph of the interior lingual glands showing neutral mucin reaction in the lateral group (LG) and both neutral and acid mucin reactions in the medial group (MG). Alcian blue-PAS stain.

**Figure 7:**
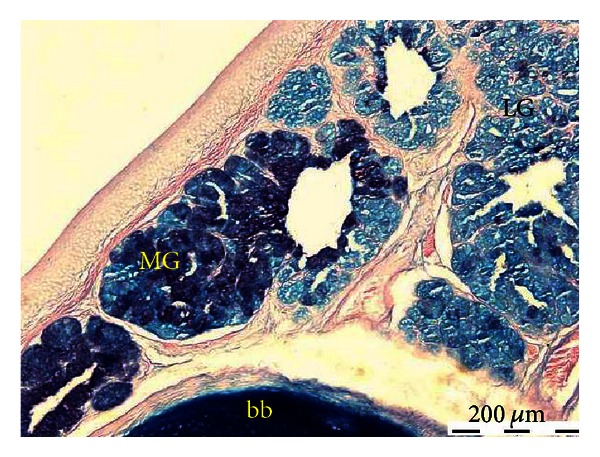
Microphotograph of the interior lingual glands of the common myna tongue, showing sulphated mucin reaction in the lateral group (LG) and both sulphated and carboxylated mucin reactions in the medial group (MG). Base hyoid bone (bb). Aldehyde fuchsin-alcian blue stain.

In all salivary glands of the tongue, some of the mucin granules of the secretory cell reacted with PAS stain and the others reacted with alcian blue stain after alcian blue-PAS stain (Figure 6). Staining with aldehyde fuchsin-alcian blue stain, only the LG reacted with alcian blue. Meanwhile, the MG and the PLG reacted positively with both alcian blue and the aldehyde-fuchsin stain (Figure 7). 

## 4. Discussion

The tongue of birds is adapted to the route and type of food intake [7]. The conical lingual papillae of the common myna tongue appeared different in arrangement than that in red jungle fowl tongue [6] and in the common kestrel [8]; in these species, the conical papillae are arranged in a transverse row. In goose, these papillae are restricted in the midline between the body and the base of the tongue [15] or restricted in a single crest which extended from the body to the root of the eagle tongue [4]. The caudal directed papillae facilitate the prehension and swallowing of food [8]. Similar to the present study, the pharyngeal papillae of the red jungle fowl appear as a single row [6]. However, there are two rows of the pharyngeal papillae in fowl [16].

In the present study, the tongue was covered by parakeratinized stratified squamous epithelium. This was strongly keratinized on the ventrolateral surface and the lingual papillae. Similar findings were obtained in different species of birds. However, the degree of keratinization of the epithelium depended on the type of food intake; in herbivorous and granivorous birds it is appeared heavily cornified [5]. Lesser degree of keratinization is found in water habitats birds [9, 10]. In the current study, the lingual salivary glands consisted exclusively of mucus. However, Rossi et al. [11] have shown similar results in partridge. Similar to the present study, the anterior and the posterior lingual glands of the red jungle fowl have some difference after histological stain [6]. In contrast, there are no lingual salivary glands in cormorants [10].

The histochemical observations of the current study revealed the presence of a large amount of secretory granules in the cell cytoplasm of the MG and the PLG compared with the LG of the ALG after PAS stain. This indicated that the glycoconjugates contained vicinal diol group. Similar results were reported in red jungle fowl [6], whereas, the lingual salivary glands of the little egret were considered free of neutral mucosubstance [12]. Subsequent to alcian blue- PAS stain, the LG of the ALG of the common myna tongue contained neutral mucin. Meanwhile, the MG of the ALG and the PLG contained both neutral and acid mucin. However, these results seem similar to that founded in chicken [17] and in red jungle fowl [6]. The results of current study showed different reactions with alcian blue stain when the PH changed indicating that the LG contained weak acid mucin, and the MG and the PLG had strong acid mucin with the carboxylated and sulphated groups. Meanwhile, the LG had carboxylated mucin only. However, similar results to these data were reported by Gargiulo et al. [18] in chicken, in the little egret [12], and in red jungle fowl [6].

## 5. Conclusion

The tongue of common myna appeared similar to the other birds; however, some differences were found regarding the arrangement of the lingual and pharyngeal papillae. The nature of the neutral mucin of the lingual salivary glands may act as lubricant of food to facilitate swallowing. In addition, the mucin preserves hydration by providing a hydrophilic environment. Moreover, Slomiany et al. [19] reported that the acid mucin plays a role in the modulation of the oral calcium channel activity.
